# 

**Published:** 1874-12

**Authors:** 


Vol. IV. DECEMBER, 1874. No. 12.

### THE SOUTHERN MEDICAL RECORD:

A MONTHLY JOURNAL OF

### PRACTICAL MEDICINE.

EDITORS:

T. S. POWELL, M.D., - - - - - Atlanta, Georgia.
W. T. GOLDSMITH, M,D., - - - - Atlanta, Georgia.
T. CURTIS SMITH, M.D., - - - - Middleport, Ohio.
SWAN M. BURNETT, M.D., - - - Knoxville, Tennessee.
ALBAN S. PAYNE, M.D., - - - - Markham's, Virginia.
A. R. KILPATRICK, M.D., - - - Navasota, Texas.
A. A. LYON, M.D., - - - - Columbus, Mississippi.
R. C. WORD, M.D., - - - - - Decatur, Georgia.

"*QUICQUID PRÆCIPIES ESTO BREVIS.*"

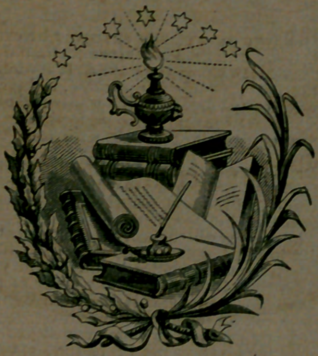

ATLANTA, GEORGIA:
SOUTHERN PUBLISHING COMPANY, PRINTERS.
1874.

Terms, $3 00 Per Annum, in Advance. Single Number, 25 Cents.

